# Inflammation and Inflammasomes: Pros and Cons in Tumorigenesis

**DOI:** 10.1155/2020/2549763

**Published:** 2020-09-19

**Authors:** Liliana R. Balahura, Aida Selaru, Sorina Dinescu, Marieta Costache

**Affiliations:** ^1^Department of Immunology, National Institute for Research and Development in Biomedical Pathology and Biomedical Sciences “Victor Babes”, Bucharest 050096, Romania; ^2^Department of Biochemistry and Molecular Biology, Faculty of Biology, University of Bucharest, Bucharest 050095, Romania; ^3^The Research Institute of the University of Bucharest, Bucharest 050663, Romania

## Abstract

Over the past decade, it has been well established that tumorigenesis is affected by chronic inflammation. During this event, proinflammatory cytokines are produced by numerous types of cells, such as fibroblasts, endothelial cells, macrophages, and tumor cells, and are able to promote the initiation, progression, and metastasis of different types of cancer. When persistent inflammation occurs, activation of inflammasome complexes is initiated, leading to its assembly and further activation of caspase, production of proinflammatory cytokines, and pyroptosis induction. The function of this multiprotein complex is not only to reassure inflammation and to promote cell death, through caspase activity, but also has been identified to have significant contributions during tumorigenesis and cancer development. So far, many efforts have been made in order to extend the knowledge of inflammasome implications and how its components could be targeted as therapeutic agents. Additionally, microRNAs (miRNAs), evolutionary conserved noncoding molecules, have emerged as pivotal players during numerous biological events by regulating gene and protein expression. Therefore, dysregulations of miRNA expressions have been correlated with inflammation during tumor development. In this review, we aim to highlight the dual role of inflammasomes and proinflammatory cytokines during carcinogenesis paired with the distinguished effects of miRNAs upon inflammation cascades during tumor growth and progression.

## 1. Introduction

Inflammation represents an immune response of the host to damaging stimulation, realized by pathogens or irritants. Persistent inflammation is a favorable factor for the development of chronic diseases, such as autoinflammatory or autoimmune disorders, neurodegenerative diseases, metabolic conditions, and cancer [1].

The inflammatory cascade is one of the factors responsible for cytokine and chemokine activation [2]. Cytokines are small-sized proteins with important functions in cellular interactions, which are classified depending on their cellular origin and characteristics in lymphokines, monokines, chemokines, and interleukins [3]. Their mechanism of action can be managed in an autocrine, paracrine, or endocrine manner, cytokines being involved in homeostasis and host defense [4]. Chemokines are proteins with cysteine radicals in their structures, capable of chemotaxis induction [3]. There are two main types of chemokines: the first type has a homeostatic role and the second type of chemokines is secreted by cells after proinflammatory stimulus recognition [5].

The most studied cytokine is interleukin- (IL-) 1*β*, which is secreted by various types of cells, such as monocytes, macrophages, fibroblasts, or endothelial cells [3] in different pathological conditions (inflammation, infection, invasion, tissue damage, or cancer) [6]. Another cause of cytokine and chemokine secretion (IL-1*β* and IL-18) is represented by caspase-1 activation due to conformational modifications caused by the inflammasome complex [7].

Inflammasomes are defined as high-molecular-weight cytoplasmic multiprotein complexes, which have been described mostly on epithelial cells due to their propagation properties in *in vitro* cultures [8]. Further, the theory that chronic inflammation together with reactive oxygen species production is responsible for DNA damage leading to cancer evolution [9] is well accepted. Therefore, inflammasome hallmark in tumor growth and progress is a hot topic among researches and has been widely explored in the last decade [10].

The development of chronic stage is sustained by the presence of cytokines and chemokines at a high level of expression [11]. For example, tumor-associated macrophages (TAMs) are derived from monocytes, being present in neoplastic tissues. TAM functions are crucial in tumorigenesis progression, intervening in secretion of numerous cytokines, proteases, and angiogenic and lymphangiogenic growth factors [12], but on the other hand, they can also influence tumor cell death through IL-2, IL-12 expression, and interferon (IFN) activation [13].

Tumor development is an event which is influenced by inflammatory cells, which release reactive molecules affecting genetic information by producing genomic changes, such as mutations, rearrangements, and deletions [14]. In early stages of tumorigenesis, these cells fulfill the role of promoters being involved in genomic instability, angiogenesis, growth, migration, and differentiation of cellular components from the tumor microenvironment [15].

Inflammation and cancer are correlated through two distinct mechanisms: (1) the dysregulation in anti-inflammatory cytokine secretion and (2) the modification of host response due to high concentration of proinflammatory cytokines and chemokines [15]. The inflammatory mechanism is guided by the inflammasome machinery. The role of inflammasome complexes is to mediate the process of host defense [16]; therefore, any alteration that occurs in the activation mechanism of the complex may lead to initiation of tumorigenesis, the appearance of autoimmune diseases, neurodegenerative diseases, or metabolic disorders [17].

## 2. Inflammasome Complexes: Architecture, Classification, and Mechanisms of Activation

### 2.1. The Architecture of Inflammasomes

The inflammasome complexes are formed of three important molecular structures: a sensor molecule, an adaptor protein, and an effector, such as caspase-1. The sensor molecules that have been identified so far to take part in the assembly process are the nucleotide-binding oligomerization domain (NOD), leucine-rich repeat (LLR), NOD-like receptors (NLRs) family, absent in melanoma 2- (AIM2-) like receptors (ALRs) family, interferon-inducible protein 16 (IFI-16), and retinoic acid-inducible gene I (RIG-I). Once activated, the sensor molecules promote the involvement of the apoptosis-associated speck-like protein containing a caspase activation and recruitment domain (CARD) (ASC), which is directly involved in activation of a specific caspase [18].

All protein components of NLR inflammasomes are present in their structure death domains (DD), represented by pyrin domains (PYD) (NLRP) or CARD domains (NLRC), which have specific functions in the complex assembly [19]. Being part of the NLR family, NLRP1, 3, 4, 6, 7 and NLRC12 are capable of inflammasome complex formation [18]. After receptor activation, the downstream assembly of ASC is stimulated to develop a speck structure or protein aggregate restricted in the paranuclear cell area, which leads to maturation of procaspase-1 and then further to pro-IL-1*β* and pro-IL-18 maturation [20].

### 2.2. Components of Inflammasome Complexes

Inflammasome complexes assemble three major molecular elements: (1) pattern recognition receptors (PRRs) family serving as sensors, (2) the adaptor protein ASC, also noted as PYCARD, and (3) the caspase family [2].

PRR family compresses receptors with different cellular localizations: Toll-like receptors (TLRs) and C-type lectin (CTL), which are membrane-bound receptors and cytoplasmic receptors such as NLRs, RIG-I-like receptors (RLRs), and ALRs [21].

The adaptor protein ASC activity is based on the two protein-protein interaction domains, namely, a N-terminal PYD domain and a C-terminal CARD domain. The function of this ASC protein is connected with the complex's assembly involved in inflammatory processes and cell death [22].

Caspase family can be divided depending on their involvement in inflammation or apoptosis: inflammatory caspases (caspase-1, caspase-4, and caspase-5), caspases involved in apoptosis initiation (caspase-2, caspase-8, caspase-9, and caspase-10), and caspases required for apoptosis advancement (caspase-3, caspase-6, and caspase-7). The structural similarity between all these caspases is represented by the C-terminal protease domain (PD), although several caspases are present in their prodomain structure [23].

### 2.3. Inflammasome Classification

**Figure 1:**
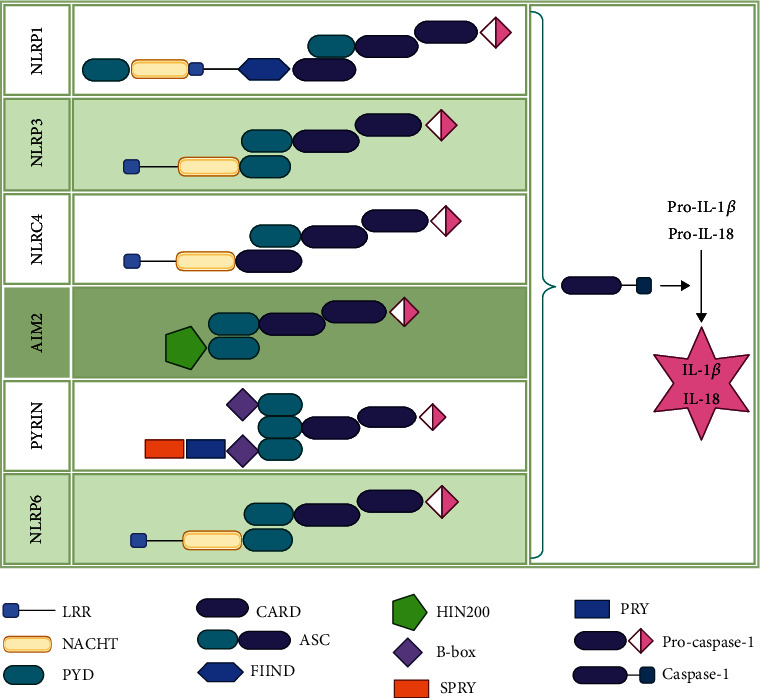
Particular structures of inflammasome complexes composed of three main components: sensor, adaptor, and effector. The NLR proteins (Nlrp1, Nlrp3, Nlrp6, and Nlrc4) and HIN200 protein AIM2 mobilize the complexes in a stimulus-specific manner. Activated receptors recruit the ASC adaptor protein, and the CARD domain from ASC protein structure is necessary for procaspase-1 recruitment and caspase-1 activation. Active caspase-1 induces secretion and maturation of pro-IL-1*β* and pro-IL-18 into their active forms IL-1*β* and IL-18 (image created in https://BioRender.com).

Over the years, several different inflammasome complexes (Figure 1) have been identified according to the proteins responsible for the signaling pathway initiation [8]. Because of the high versatility in molecular patterns, expression, and stimulus motifs, inflammasomes present various protein structures, which imply different ways of assembly and action [24]. Therefore, we will further separately describe each type of inflammasome and its assembly process.

#### 2.3.1. The NLRP1 Inflammasome

The NLRP1 inflammasome complex is composed of a NOD, LRRs, an amino-terminal PYD domain-containing protein 1 (NLRP1), a carboxy-terminal domain CARD, and a function-to-find domain (FIIND) [17].

The assembly and activation of the NLRP1 inflammasome are determined in a signal-specific way, which is represented by the N-terminal proteolytic cleavage [25]. Any disorder of the functioning of this type in NLRP1 inflammasome can lead to skin disorders, different types of carcinoma, and chronic diseases [26].

### 2.4. The NLRP3 Inflammasome or Cryopyrin

The NLRP3 is the most studied and best characterized type of inflammasome. Its activation is stimulated by a wide range of extracellular stimuli, such as uric acid crystals, silica, asbestos alum, adenosine triphosphate (ATP), and some pathogens [18]. After the signals are recognized by the specific receptors, the transcriptional initiation of NLRP3 inflammasome complex is stimulated and different mechanisms emerge, such as potassium efflux, reactive oxygen species (ROS) production, mitochondrial and lysosomal dysfunctions, appearance of pores, or oxidized mitochondrial DNA [18, 27].

Activation of NLRP3 inflammasome requires two specific signals, priming and assembly. Priming signal depends on activation of myeloid differentiation primary response protein (MyD88), nuclear factor kappa-light-chain-enhancer of activated B cells (NF-*κ*B), or the activator protein-1 (AP-1) signaling pathways. The assembly of the NLRP3 complex counts on extracellular ligand recognition signals [28].

#### 2.4.1. The NLRC4 Inflammasome

It has been discovered that NLRC4 is receptive to bacterial type 3 secretion systems (T3SSs). Therefore, upon detection of bacterial flagellin, activation of NLRC4 inflammasome occurs [29, 30]. To initiate its assembly, receptors from NLR family, specifically apoptosis inhibitory proteins (NAIPs), are necessary. NAIPs are able to detect T3SSs, thus leading to structural changes paired with recruitment and oligomerization of NLRC4 [31].

For initiation of complex assembly and NLRC4 oligomerization, only one activated NAIP molecule is necessary [32]. Further, NLRC4 becomes an adaptor and is responsible for caspase-1 recruitment [33]. This connection between NLRC4 and NAIPs is beneficial for host defense, maturation of IL-1*β* and IL-18, and eicosanoid release [16].

#### 2.4.2. The AIM2 Inflammasome

AIM2 possesses a carboxy-terminal HIN-200 (hematopoietic expression, interferon-inducible nature, and nuclear localization) domain, which mediates a direct interaction with dsDNA and an amino-terminal PYD domain which binds to the ASC adaptor protein via CARD [34]. AIM2 inflammasome activation develops upon the cooperation between bacterial lysates [35] and PYD and HIN domains, thus triggering the assembly of the complex [36].

According to several studies [37, 38], it is suggested that initiation and progression of tumorigenesis are also influenced by the low levels of AIM2. A possible explanation for this theory is detection of self-DNA, specifically in prostate [37] and colorectal cancer [38].

#### 2.4.3. The Pyrin Inflammasome

Pyrin (also known as marenostrin or TRIM20 receptors) represents a group of receptors capable of initiating the assembly and activation of inflammasomes. The characteristic structure of a pyrin receptor consists of a PYD domain, two B-boxes, a coiled-coil domain, and a C-terminal SPRY/PRY domain [17].

Assembly of the pyrin inflammasome takes place upon the covalent changes, such as glycosylation, adenylation, or ADP-ribosylation, in the structure of cytoskeletal proteins, specifically proteins of Rho family [39].

#### 2.4.4. The NLRP6 Inflammasome

The NLRP6 pathway is a less well-characterized mechanism, but its involvement in development and progression of colitis has been established. This may lead to the initiation of tumorigenesis, as a result of abnormal secretion of IL-18 by intestinal epithelial cells [40].

This type of inflammasome needs to be further studied in detail, but so far, it is known that NLRP6 complex influences IL-18 and goblet cell mucus secretion [41], regulates NF-*κ*B and the mitogen-activated protein kinase (MAPK) signaling pathways [42], and controls the production of interferons type I and III [43].

## 3. Mechanisms of Activation

**Figure 2:**
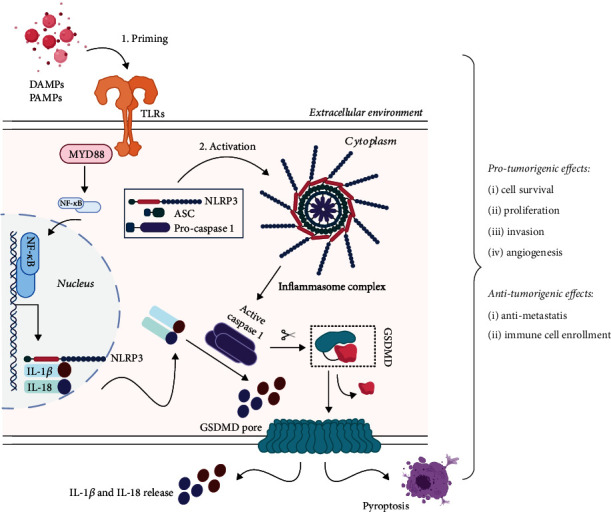
The canonical pathway and activation mechanism of inflammasomes and outcomes in cancer. PAMP and DAMP signals are recognized by PRRs/TLRs, which then lead to activation of caspases. Active caspase-1 will promote the IL-*β* and IL-18 release or pyroptosis initiation, through GSDMD cleavage and pore formation (image created in https://BioRender.com).

Specific sensors, such as PRRs or TLRs, are able to recognize distinct stimuli represented by pathogen-associated molecular patterns (PAMPs) and damage-associated molecular patterns (DAMPs). As stated before, inflammasomes present fundamental structural differences, thus leading to opposed mechanism of action upon stimulation, but generally, canonical inflammasomes serve as a platform to enroll inactive procaspase-1 [44]. Upon sensing of PAMPs and DAMPs, receptor molecules will oligomerize and will further generate the activation of caspase-1 [45] (Figure 2). Activated receptor molecules engage with the adaptor protein ASC, which recruits procaspase-1 and determines its activation through oligomerization and autoproteolysis. Afterwards, the active caspase-1 supports the cleavage and maturation of pro-IL-*β* and pro-IL-18, which further promote the secretion of other inflammatory factors and lead to pyroptosis [23, 46].

Two noncanonical pathways of inflammasome activation have been identified, which involve other caspases than caspase-1. The first one considers the capacity of lipopolysaccharides (LPS) to activate caspase-4 and caspase-5 through TLR4 receptors, which will further influence the processing of gasdermin D (GSDMD) in order to activate the inflammasome complex. After activation of inflammasome, IL-1*β* and IL-18 maturation will be realized in a caspase-1-dependent manner and the process of pyroptosis will be promoted [47]. An alternative to noncanonical activation pathway is represented by IL-1*β* and IL-18 maturation through activation of caspase-8, which is activated through microbes' detection by CTL receptors [48].

## 4. Effectors of the Inflammasome Complexes and Their Implications in Cancer Pathogenesis: IL-1*β* and IL-18 Signaling

Over the years, the connections between inflammation and cancer have been permanently studied. Interleukins are part of cytokine family and represent secreted proteins which bind to their specific receptors, being involved in immune activity and tumorigenesis [49]. IL-1*β* is part of IL-1 family, which is translated as pro-IL-1*β* and maturated through caspase-1 activity, and it can bind IL-1 type I receptor (IL-1RI) or IL-1 type II receptor (IL-1RII). IL-1*β* targets different types of cells, such as T cells, epithelial cells, fibroblasts, or endothelial cells, contributing to hematopoiesis or stimulation of proinflammatory proteins [50]. Its activity can be correlated with inflammatory diseases, autoimmune diseases, inflammatory bowel disease, rheumatoid arthritis, psoriasis, or cancer [51].

Another member of IL-1 family is IL-18, a heterodimer which depends on caspase-1 activity to be maturated. The target cells of IL-18 are partly similar to those targeted by IL-1*β*, particularly T cells, epithelial cells, macrophages, and natural killer (NK) cells [52]. Its functions are to increase NK cell toxicity and to induce secretion of IFN-*γ* together with IL-12, and its activity can be associated with numerous disorders, such as inflammatory diseases, autoimmune diseases, type I diabetes, Crohn's diseases, multiple sclerosis, and tumor development [53].

High levels of cytokines, especially IL-1*β* and IL-18, were detected in the tumor microenvironment [54]. Their presence influenced inflammation, premalignant cell proliferation, and angiogenesis, and promoted tumor initiation and progression and metastasis development [49].

**Figure 3:**
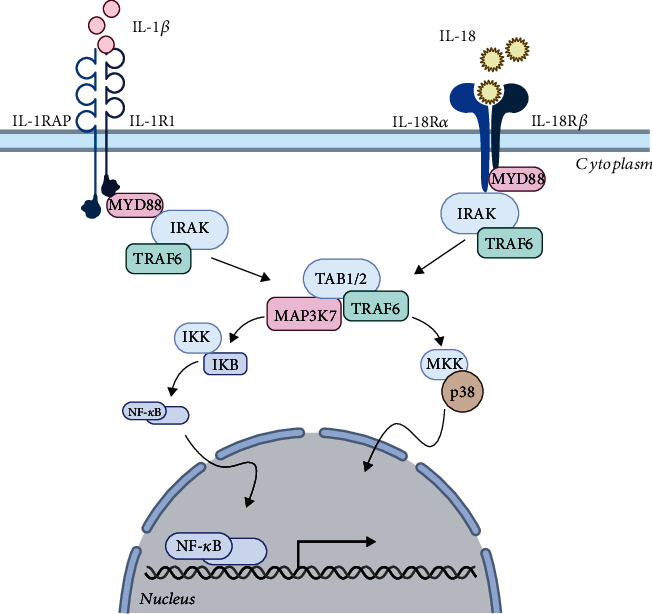
Signaling pathway of IL-1*β* and IL-18. IL-1*β* molecules are released in the cytosol and bind to IL-1R1 receptor in order to form a heterodimer with IL-1RAP. IL-1R is phosphorylated by MyD88 and IRAKs; thus, TRAF-6 are activated and able to further activate specific MAP kinases and NF-*κ*B. Then, NF-*κ*B is translocated to the nucleus and initiates the transcription of proinflammatory molecules. Similar to IL-1*β*, the IL-18 molecules follow a parallel pathway with the same outcome of production of proinflammatory molecules (image created in https://BioRender.com).

As IL-1*β* is not found to be expressed in homeostatic conditions, its elevated expression can be noticed in many types of solid tumors, such as colon cancer [55], breast cancer [56], or melanomas [57], and is known as the “gatekeeper” of inflammation [58]. IL-1*β* is known to be the most pyrogenic molecule in the human body. Upon the stimulation of PAMPs (Figure 3), IL-1*β* is synthesized, but its activation is connected to a secondary stimulus, the DAMPs. As soon as IL-1*β* molecules are formed and processed by inflammasomes, they are released in the cytosol and they bind to IL-1RI in order to form a heterodimer with IL-1R accessory protein. All these events lead to the triggering of the IL-1 signaling pathway. Next, IL-11R is phosphorylated by an intracellular adaptor protein, MyD88, with the help of its cytoplasmic Toll/IL-1 receptor (TIR) domain. In this manner, the interleukin-1 receptor-associated kinases (IRAKs) and TNF receptor-associated factor- (TRAF-) 6 are activated and are able to further activate specific MAP kinases and NF-*κ*B. Later, NF-*κ*B is translocated to the nucleus and initiates the transcription of multiple proinflammatory molecules; among them are IL-6, tumor necrosis factor alpha (TNF*α*), vascular endothelial growth factor (VEGF), and inducible nitric oxide synthase [59].

Similar to IL-1*β*, the mature bioactive molecule of IL-18 (also known as INF-*γ* inducing factor) is generated from an inactive peptide, which becomes active when the proteolytic cleavage by caspase-1 occurs in the NLRP3 inflammasome. The IL-18 receptor (IL-18R1) is formed of two components (an inducible one, IL-18Ra, and a constitutively expressed component, IL-18Rb) and is responsible for the activation of the IL-18 signaling pathway, which is very similar to the IL-1*β* one [60].

## 5. Molecular Inflammatory Events during Cancer Development and Progression

### 5.1. Inflammasome Involvement in Cancer Development and Progression

Since the inflammasomes' discovery, researchers have concentrated their work in finding its function and contribution to cancer progression. Various published science papers [61–66] have illustrated different types of inflammasome behavior and dynamic functioning during cancer development, migration, and metastasis (Table 1).

Among all types of inflammasomes, NLRP3 has caught the attention of many research groups and presented tremendous interest due to its involvement in various types of tumor development [71]. The function of NLRP3 during tumorigenesis is still controversial, while some studies evidenced its protective role, and others came to the conclusion that its action has destructive outcomes, whereas, in head and neck cancer [72], the activation of cryopyrin leads to tumor outgrowth, and in colitis-associated tumor, secretion of IL-18 via NLRP3 delivered protection against malignant tissue development [73]. Ershaid et al. [68] investigated the correlation between NLRP3 inflammasome activation in fibroblast and its effect upon breast cancer development and metastasis. Their results indicated an upregulation of the NLRP3 pathway for both murine mammary carcinogenesis and cancer-associated fibroblasts in human breast cancer conditions. Further investigations have led to the conclusion that NLRP3-mediated inflammation is responsible for tissue damage in the case of breast cancer. Interestingly, Kumar et al. [69] explored different epigenetic modifications which could be held responsible for NLRP3 expression during renal cancer. In their study, they have found high expression levels of NLRP3 pathway components in clear cell renal cell carcinoma patient samples as compared to the experiment's controls, thus suggesting a crucial role of NLRP3 inflammasome in this type of tumor. Further in-depth molecular analysis highlighted an impressive regulation of lysine-specific demethylase 2 upon the NLRP3 pathway, leading to the conclusion that posttranslational histone modifications may have a significant contribution for NLRP3-mediated inflammation for renal tumorigenesis.

Besides NLRP3, other types of inflammasomes have been identified in playing specific roles in tumor progression and metastasis. In this context, Zhai et al. [70] have built up a complex experiment in order to establish the biological functions of NLRP1 inflammasome in both *in vitro* and *in vivo* melanoma conditions. They have found that NLRP1 was mainly expressed in the cell cytoplasm and that knocking NLRP1 down leads to tumor-promoting events. Moreover, the lack of NLRP1 leads to reduced activity of caspase-1, IL-1*β*, and NF-*κ*B in various human melanoma cell lines. Additional findings of this study lead to the conclusion that NLRP1 possessed tumor-enhancing role in melanoma cells.

Caspases are proteolytic enzymes which have the ability to regulate the process of inflammation and apoptosis [74]. Up to date, a certain number of mammalian caspases have been found to be crucial mediators of the innate immune response and are known as inflammatory caspases [75]. Besides, it is well established that uncontrolled cell death is a hallmark of tumorigenesis and recent investigations have liked the aberrant expression of apoptotic caspase to cancer migration outgrowth [76]. In contrast, Ho et al. and Krelin et al. suggested that some caspases may have tumor suppressor characteristics [77, 78]. Another interesting study involved in the caspase-1, caspase-3, and caspase-9 protein expressions was realized by Winter and his collaborators. They observed that in the case of prostate cancer, the caspase cascade is dysregulated, because the expressions of caspase-1 and caspase-3 were decreased and the mRNA expressions have not been significantly modified. The conclusion of this study was that prostate cancer cells display loss of key caspase expression, which confers stability against apoptosis to the tumor cells [79]. To sum up, all these data present a clear dual role of inflammasomes during cancer events. There is no defined role that the inflammasome possesses in the case of tumors; its function depends not only on the stage and type of cancer but also on the body's capability of each patient.

### 5.2. Cytokine Involvement in Cancer Development and Progression

Cytokines are glycoproteins or polypeptides which have a distinguished function, mainly to ensure anti- or proinflammatory signals in normal and pathological conditions. Mostly, these molecules are released during a certain time upon signal stimulation and the period of their action is most likely a reduced one, because of their limited life span in circulation [80]. Being part of the cytokine family, IL-1 family has been identified as a class of molecules, which mediate cellular communication within the immune system, possess multiple immunomodulatory functions, and participate in the regulation of tumor microenvironment [81]. Among them, IL-1*β* and IL-18 are proinflammatory cytokines [82] and their actions during carcinogenesis development and progression will be further discussed.

Because of its pleiotropic nature, IL-1*β* affects the activity of multiple cell types; thus, it was found to increase vascular permeability and the expression of adhesion molecules and matrix metalloproteinases. Due to these actions, IL-1*β* grants a barrier against microbial infections by killing different species of pathogens. Yet, this particular molecule and its mechanism of action can be held responsible for tumor cell migration, proliferation, and metastasis [83]. Up to date, lots of evidence in both *in vitro* and *in vivo* experiments suggest that IL-1*β* possesses a promoting effect on cancer development and progression [84]. Carmi et al. [85] have demonstrated in their study the interplay between IL-1*β* and VEGF, namely, that IL-1*β* is produced by myeloid cells, which will lead to the production of VEGF by endothelial cells, thus providing an inflammatory microenvironment for angiogenesis and tumor progression. Moreover, other studies [86] have shown how IL-1*β* is responsible for enhancing tumor metastasis. In this context, Guo *et al*. [64] generated a murine breast cancer model and found that tumor progression was associated with inflammasome activation and high levels of IL-1*β* at metastatic sites. On top of that, they demonstrated that mice deficient for inflammasome components have presented significantly reduced lung metastasis. Therefore, the fact that IL-1*β* is inducing the production of protumorigenic molecules is a central event during cancer progression. Recently, another important link between IL-1*β* and a protumorigenic element has come to light. It seems that the production of IL-22 is dependent on the activation of NLRP3 inflammasome with the result of IL-1*β* secretion from both myeloid and T cells [87]. All these data suggest that IL-1*β* is a pivotal contributor to cancer development and has the potential to be validated as a therapeutic target in the constant fight against cancer.

While IL-1*β* is expressed only in pathological conditions, IL-18 expressions are also found in healthy human blood monocytes and epithelial cells [88]. Due to IL-18 capability of inducing IFN-*γ* production in T, B, and NK cells, it is a significant component of inflammatory processes. All studies involved in understanding the IL-18 mechanism of action have investigated both anti-ancerous and procancerous effects of this cytokine with the possibility of using it for therapeutic processes. The role of IL-18 in cancer progression, metastasis, and angiogenesis still remains controversial, but its secretion has a particular contribution to tumor environment regulation [89, 90]. This multifunctional cytokine has a dual effect on tumor cells and is explained by researchers as a double-edged sword [91]. The antitumor effect of IL-18 is mostly due to IFN-*γ* production in developing immune responses. While administration of IL-18 is responsible for tumor regression in animal cancer models [92], the same cytokine found in high serum concentrations, in some cancer types, demonstrates its protumor effects [89]. Among the first researchers that have investigated this matter, Park et al. [93] have observed multiple effects of IL-18 in several melanoma cell types. With the help of the enzyme-linked immunosorbent assay (ELISA) technique, they have found high expressions of IL-18, thus suggesting the involvement of this cytokine in cancer growth. Moreover, studies illustrate the contribution of IL-18 during tumor metastasis development. In this context, Zhang *et al*. [94] demonstrated that IL-18 succeeded in inhibiting hepatitis B virus (HBV) in various liver cell lines. At the same time, they observed that IL-18 promoted hepatoma cell metastasis and migration, revealing the dual effects of IL-18. It can be concluded that IL-1*β* and IL-18 activities are modulated by their concentrations in the tissue, by the expression profiles of their specific receptors, and by their specific inhibitors [4]. In normal conditions, the role of IL-1*β* is to stimulate IL-6 and IL-17a release and IL-18 role is to promote IFN-*γ*, IL-2, and IL-12 production [95]. Even so, under specific stimulation, they present a dual behavior. The reasons behind the switch in function of IL-1*β* and IL-18 still remain to be elucidated. More research is necessary in finding how these cytokines can be targeted in order to contribute to cancer regression.

### 5.3. Oxidative DNA Damage Involvement in Cancer Development

The imbalance between ROS production and antioxidant mechanisms leads to oxidative stress appearance. Oxidative stress is one of the factors involved in genomic instability, inflammation, and cancer, due to the action on proteins, lipids, and nucleic acids. Oxidative DNA damage is one of the amplifying factors of the DNA-repair machinery burden [96].

ROS are resulted from oxidative metabolism and are responsible for the defects in the DNA repair mechanism. 8-hydroxy-2-deoxyguanosine is a product of DNA oxidation, which is responsible for the mutations in the DNA and for the development and progression of carcinogenesis [97].

ROS involvement in DNA damage is proven through nucleoside base oxidation and by forming different compounds, such as 8-oxo guanine. Enormous ROS production causes oxidative stress and destructive effects on the mitochondria, such as membrane integrity or membrane potential damage. Under ROS influence, DNA oxidation leads to 8-hydroxy-2-deoxyguanosine formation, which is responsible for the occurrence of DNA mutations and chronic diseases. Due to its low redox characteristics, the base guanine (G) is exposed to oxidation and results oxidized G products, which produce DNA lesions. One of the lesions is represented by 7,8-dihydro-8-oxo-2′-deoxy-guanine (8-oxo-G), which is a biomarker of oxidative stress and different human diseases such as cancer. 8-oxo-G is able to mimic a thymine (T) in syn conformation and to form a stable promutagenic A (anti) : 8-oxo-G (syn) mispair [98]. During the cellular S phase, the replicative DNA polymerases insert an incorrect base, due to the stability of A : 8-oxo-G. If the proofreading activity does not correct the mispair, one of the daughter cells resulted from DNA replication will present a DNA template with a C (cytosine) : G → A (adenine) : T transversion mutation [99].

Salehi et al. studied the effects of oxidative DNA damage determined by ROS production and the effects of citrus pectin and apple pectin on human breast cancer cells MDA-MB-231, MCF-7, and T47D viability. Consequent to citrus pectin and apple pectin treatment, ROS production increased, mitochondrial transmembrane potential was affected, and cancer cells were arrested at the S and G1 or G2/M phases of the cell cycle. Some observations were that cancer cells treated with the proposed treatment produced oxidation and strand breaks in the DNA and that growth inhibition of cancer cells by citrus pectin and apple pectin treatment was concomitant with DNA damage [100].

Mertz et al. studied the incidence of mutations caused by *POLD1-R689W* and its involvement in tumor development and progression. An observation was that the preponderance of mutations induced by the *POLD1-R689W* was GC → TA transversions and GC → AT transitions. In this study, two colorectal adenocarcinoma cell lines DLD-1 and HCT-15 were employed, and it has been suggested that at their level, the expression of *POLD1-R689W* is completely mutagenic. The interesting results highlighted that 84% of base substitutions were GC → TA transversions or GC → AT transitions and that the C-deoxythymidine triphosphate (dTTP) is one of the mispairs that could lead to GC → TA transversions [101].

Pallem et al. studied the frequency of mutation in colon epithelial cells in the presence of phytate and observed the evolution of O^6^-methyl guanosine and 8-hydroxy deoxyguanosine adducts. The results revealed that phytate inhibited aberrant crypt focus development, but the control indicated GGT to GAT transition, GGT to GTT transversion at codon 12, and GGC to CGC transversion at codon 13 [102].

### 5.4. Therapeutic Approaches: Targeting Inflammasomes and Their Components Means Indirectly Targeting Cancer?

Human diseases present provocative characteristics; therefore, effective treatments and therapies against cancer initiation, progression, or metastasis are currently investigated at a large scale. When it comes to cancer, more and more focus is placed in targeting the inflammatory component as potential treatment. As a result, the fact that inflammasomes and their components present a therapeutic interest, it should not come as a surprise. If an excessive activation of inflammasomes occurs, it will lead to harmful results against multiple types of cancer. Up to date, more than 50 therapeutic candidates which target component of the inflammasome signaling cascade have been proposed or even launched [103]. Among them, Anakinra, an IL-1 receptor inhibitor, is widely used for treatment of numerous pathologies, including several types of cancer [104]. Besides Anakinra, a whole other inhibitor agent is used or tested in order to find the optimal target to obtain satisfying results. Simvastatin is a statin which is known to be an inhibitor of cell proliferation *in vitro* and tumor growth *in vivo* [105]. One study realized by Wang et al. [106] investigated the influence of simvastatin over non-small-cell lung cancer (NSCLC) line. They researched the connection between simvastatin and pyroptosis initiation in lung cancer cells. To distinguish pyroptosis from apoptosis, the researchers employed western blotting to establish caspase-1, IL-1*β*, and IL-18 profiles and TUNEL assay to observe DNA fragmentation status. The results indicated that expressions of caspase-1, IL-1*β*, and IL-18 were downregulated in tumor samples and that simvastatin determined the decrease of tumor cell viability directly proportional to the dose administered, thus decreasing lung cancer cell proliferation and migration via pyroptosis initiation. Also, Zou et al. [107] demonstrated the antitumor effect of polydatin on non-small lung cancer cells A549 and H1299 cells. They observed that polydatin repressed the proliferation of lung cancer cells, evolution of lung cancer through inhibition of the NLRP3 inflammasome signaling pathway, and downregulated the activation of the NF-*κ*B signaling pathway.

Further, Yu and his team [108] tested the ability of lobaplatin to induce pyroptosis in colon cancer cells. Lobaplatin effects against tumor cells were convincing, implying decrease of tumor cell viability, cell membrane damaging, and increased IL-1*β* levels. Another observation of this study was that GSDME, which is involved in inhibiting the growth of tumor cells, regulated pyroptosis downstream of the ROS/JNK/Bax pathway. But also, classical therapeutic agents such as 5-fluorouracil have positive outcomes in NLRP3 inflammasome action. Moreover, in combination with some IL-1*β* inhibitor therapies, they provide a decrease in lethal toxicity [109]. Yao et al. [110] studied the antitumor activity and anti-inflammatory potential of berberine against breast cancer cell MDA-MB 231. The results indicated that berberine determined the decrease of cancer cell viability and proinflammatory cytokine production and downregulation of P2X purinoceptor 7 (P2X7) expression, procaspase-1, and IL-1*β*.

Even with all these evidence and studies, a drug that is capable to target inflammasomes directly has still not been reported. Due to the complexity of the inflammasome signaling pathways which have yet not been fully elucidated, researchers are facing limitations in drug discovery.

### 5.5. MicroRNAs: Mediators of Inflammation-Induced Tumorigenesis

miRNAs represent noncoding molecules of 18-25 nucleotides [111], which pair to the 3′-untranslated region (3′-UTR) of the specific messenger RNA (mRNA). miRNAs are involved in posttranscriptional gene silencing, serving for mRNA degradation and inhibition of translation [112]. Over the years, it has been established that some miRNAs can undergo aberrant expression, thus exhibiting tumor-suppressive characteristics. Moreover, the expression of miRNAs can be altered due to the inflammatory stimulation; therefore, it can be considered that miRNAs can act as mediators of inflammation-induced carcinogenesis [113].

Numerous studies [114–116] have focused on the influence of inflammatory mediators on miRNA expression, both in normal and tumor conditions. As previously discussed, cancer development is also supported by the overexpression of specific cytokines, such as TNF-*α*, IL-6, IL-1, IL-8, IL-10, IL-12, and transforming growth factor beta (TGF-*β*) [117]. These cytokines and many others are involved in the connection between the functions of miRNAs and the inflammatory responses [118].

miRNAs are simultaneously involved in inflammation, inflammatory diseases, and cancer; among them are miR-21, miR-16, miR-31, miR-155, and miR-146b [119]. miR-21 is an antiapoptotic miRNA and tumor promoter due to its involvement in cell proliferation. Moreover, miR-21 promotes cancer and was reported to be present in ulcerative colitis, colon cancer, and allergic inflammation [120, 121]. Together with miR-21, miR-181b is involved in cell transformation and is a potential regulator of the NF-*κ*B signaling pathway during vascular inflammation [122] and in the progression of leukoplakia to oral carcinoma [123]. Alluringly, it has been investigated that inflammatory signals were able to mediate chromatin alterations, which further led to an epigenetic inheritance mediated by a positive feedback loop involving NF-*κ*B transcription factor, miRNA processing factor Lin28, miR let-7, and IL-6. These findings managed to link inflammation to cell transformation during cancer [124]. miR-155 has been also identified to exhibit antiapoptotic effects, overexpressed in inflammatory conditions [125] and leukemia [126], and which is responsible for the repression of proapoptotic tumor p53-induced nuclear protein 1 [127]. On the other hand, overexpression of miR-146b alters the NF-*κ*B signaling pathway in an IL-6-dependent manner, leading to a decrease in the invasion rate of breast cancer cells [128].

Interestingly, in-depth molecular studies [129–131] concluded that noncoding molecules such as miRNA are able to inhibit the activity of inflammasomes. It was found that miRNA-223 molecule binds to the 3′-UTR of *NLRP3* mRNA and suppresses its protein expression, therefore blocking the priming and the production of IL-1*β* [132]. Besides, miRNA-155, miRNA-377, and miRNA-133a-1 also have been observed to perform similar behavior upon NLRP3 inflammasome [133].

Pan and his team studied for the first time the function of miR-23 in neuropathic pain in correlation with C-X-C chemokine receptor type 4 (CXCR-4) and its downstream signaling. The obtained results indicated that TXNIP/NLRP3 inflammasome axis is a direct downstream effector of the miR-23a/CXCR4 pathway in spinal glial cells; thus, a potential target treatment for neuropathic pain can be considered using upregulation of miR-23a to reduce peripheral nerve injury [134].

A study realized by Mearini and his team [135] examined the expression of urinary inflammasome-related miRNAs in bladder cancer, by real-time polymerase chain reaction. The results indicated increased expressions of *NLRP3*, *NLRP4*, *NLRP9*, and *NAIP* mRNAs paired with increased expression of miR-106a-5p, miR-17-5p, and miR-19a-3p compared to the control group. Following this study, the important functions of NLRs in bladder cancer have been established and the hypothesis that they regulated via miRNAs has been confirmed.

In hepatocellular carcinoma, a negative regulator of NLRP3 inflammasome, miR-223, was found to be present in high expression levels, which were associated with posttranscriptional mechanisms and proliferation of tumor cells [136]. Another miRNA which is linked with hepatocellular carcinoma and NLRP3 inflammasome is miR-30e, examined from patients' serum and proposed for diagnosis and anticancer therapeutics [137].

On the other hand, miR-223 was reported to be present in high levels and also in colorectal cancer [138]. The upregulation of miR-233 was demonstrated in several studies, using different *in vitro* experiments, with HCT116, SW620 cell lines, or other tumor cell lines or primary tissues [139–141] The overexpression of miR-233 was found to promote cancer cell proliferation and silenced FoxO3a through NF-*κ*B activation and upregulation of the inflammasome complexes [139], together with miR-22 which was also expressed but in lower levels in colorectal condition [94].

Studies realized for understanding oral squamous cell carcinoma mechanisms and revealed that overexpression of both miR-223 and miR-22 suppressed NLRP3 inflammasome and NF-*κ*B signaling through ras homolog family member B (RHOB) and were considered as potential therapy targets [142]. Similar results were obtained studying cervical cancer pathogenicity, where it was observed that overexpression of miR-223 suppressed FOXO1, reduced proliferation of tumor cells, and regulated the inflammasome complex activation through the NF-*κ*B signaling pathway [143].

Keklikoglou and the research team [144] investigated the miR-520/373 family involvement in the NF-*κ*B and TGF-*β* signaling pathway regulation and their impact on breast cancer. The results indicated tumor-suppressive activity of miR-520/272 family in breast cancer, by its linking action between the NF-*κ*B and TGF-*β* signaling pathways.

Tang et al. [130] investigated miR-233 capacity to suppress breast cancer tumor cells by NLRP3 inflammasome inactivation. Their results indicated decreased proliferation rates and increased apoptotic rates of MCF-7, employing NLRP3 knockdown associated with miR-233 involvement. Their findings could represent a therapeutic objective for breast cancer treatment as the miR-233/NLRP3-mediated cancer pathway is optimal for immunosuppression of breast cancer tumor cells.

A bioinformatic study realized by Glinsky [145] indicated that specific miRNAs can target NLRPs; thus, miR-125 was associated with NLRP1 and NLRP3, miR-181 might target NLRP8, miR-143 may be involved in NLRP1 regulation, miR-200 is involved in NLRP3 and NLRP4 regulation, and miR-520 and miR-548 can target NLRP1 and NLRP3.

The correlation between cancer and inflammation is just a beginning to be understood, and lots of cellular and molecular events remain to be deciphered. A solid apprehension of miRNA mechanism of action upon inflammatory genes during cancer development may lead to groundbreaking therapeutic outcomes in clinical applications and trials.

## 6. Conclusions

To conclude, the multiprotein complexes, called inflammasomes and their connection to cancer, have been largely explored in the last few years. As presented above, it has been clearly evidenced that inflammasomes have different types of roles in various tumor types. At the same time, their contrasting role in cancer invasion, progression, and metastasis is constantly debated. For many years now, it is clear that chronic inflammation is correlated with tumor progression. Even so, despite all the efforts put in the research so far, there still is no clear evidence of how exactly inflammasomes take part in tumor evolution. A series of more in-depth molecular studies are necessary in order to establish the concrete interaction between these two major biological processes and how it can be targeted in order to have significant results when searching for the optimal therapeutic agents. Considering the complexity of the subject and to understand the molecular mechanisms, miRNA involvement in elaboration of inflammatory response and inflammasome complexes were established, thus associating the abnormal expression of specific miRNAs with inflammatory disorders and cancer.

## Figures and Tables

**Table 1:** Correlation between the roles of various inflammasome pathway components in cancer migration, growth, and metastasis.

Components of inflammasome pathway	Role	Cancer type	Experimental models
AIM2	Enhances cell proliferation and tumor migration	Cutaneous squamous cell carcinoma (cSCC)	Surgically removed human SCC of the skin [63]
NLRP3	Supports fast cancer cell migration *in vitro* and metastatic ability *in vivo*	Colorectal cancer	Human colon cancer cell line SW480, LoVo Mouse colon cancer cell line MC38, CT26 Mouse bone marrow [65]
	Actively implicated in proliferation (*in vitro*) and metastasis (*in vivo*)	Oral squamous cell carcinoma (OSCC)	OSCC cell line Normal human oral epithelial cells Mouse xenograft model [67]
	Supports tissue damage like with tumor-promoting inflammation in cancer-associated fibroblasts	Breast carcinogenesis	Mouse stains: FVB/N-Tg MMTV-PyMT 634Mul/J transgenic mice, Nlrp3^−/−^ mice, nontransgenic FVB/n mice, Balb/c mice, and C57BL/6J mice Cell lines: Met-1 (the mouse malignant neoplasms of the mouse mammary gland), 4T1(mimic stage IV human breast cancer) [68]
	Mediates inflammation during cancer development via histone regulation	Renal cancer	Human kidney carcinoma cell line (A498) Human clear cell renal cell carcinoma confirmed histological sections [69]
ASC	Tumor-promoting effects via the NF-*κ*B signaling pathway	Gastric cancer	*gp130* ^F/F^: *Asc*^−/−^, *gp130*^F/F^: *Il1r*^−/−^, and *gp130*^F/F^: *Il18*^−/−^ stomach mouse tissue and bone marrow cells [66]
	Protumorigenic functions	Gastric cancer	Human gastric biopsies [66]
NLRP1	Promotes tumor growth and suppresses apoptotic pathways	Melanoma	THP-1 (human leukemia monocytic cells) cells and 13 human melanoma cell lines [70]

## Data Availability

No data were used to support this study. This is a review and no data sets were used in the manuscript.

## Abbreviations

3′-UTR: 3′-untranslated region
8-oxo-G 7: 8-dihydro-8-oxo-2′-deoxy-guanine
A: Adenine
AIM2: Absent in melanoma 2
ALR: AIM2-like receptor
AP-1: Activator protein 1
ASC: Apoptosis-associated speck-like protein containing a CARD
ATP: Adenosine triphosphate
C: Cytosine
CARD: Caspase activation and recruitment domain
cSCC: Cutaneous squamous cell carcinoma
CTL: C-type lectin
CXCR-4: C-X-C chemokine receptor type 4
DAMPs: Damage-associated molecular patterns
DD: Death domains
dTTP: Deoxythymidine triphosphate
ELISA: Enzyme-linked immunosorbent assay
FIIND: Function-to-find domain
G: Guanine
GSDMD: Gasdermin D
GSDME: Gasdermin E
HBV: Hepatitis B virus
HIN: Hematopoietic expression, interferon-inducible nature, and nuclear localization
HPRT: Hypoxanthine phosphoribosyltransferase
IFNs: Interferons
IFI-16: Interferon-inducible protein 16
IL: Interleukin
IL-1RI: IL-1 type I receptor
IL-1RII: IL-1 type II receptor
IL-18R1: IL-18 receptor 1
IRAKs: Interleukin-1 receptor-associated kinases
MAPK: Mitogen-activated protein kinase
MMR: Mismatch repair
mRNA: Messenger RNA
miRNA: MicroRNA
NAIPs: NLR family, apoptosis inhibitory proteins
NF-*κ*B: Nuclear factor kappa-light-chain-enhancer of activated B cells
NK: Natural killer
NLR: NOD-like receptor
NLRC: NLR family CARD domain-containing protein
NLRP: Nucleotide-binding oligomerization domain, leucine-rich repeat, and pyrin domain-containing protein
NOD: Nucleotide-binding oligomerization domain
NSCLC: Non-small-cell lung cancer
OSCC: Oral squamous cell carcinoma
P2X7: P2X purinoceptor 7
PAMPs: Pathogen-associated molecular patterns
PD: Protease domain
PRR: Pattern recognition receptors
PYD: Pyrin domain
PYHIN: Pyrin and HIN200 domain-containing proteins
RHOB: Ras homolog family member B
RIG1: Retinoic acid-inducible gene-I
RLRs: RIG1-like receptors
ROS: Reactive oxygen species
T: Thymine
TAMs: Tumor-associated macrophages
TIR: Toll/IL-1 receptor
TLRs: Toll-like receptors
TNF*α*: Tumor necrosis factor alpha
TGF: Transforming growth factor
TRAF-6: TNF receptor-associated factor 6
T3SSs: Type 3 secretion systems
VEGF: Vascular endothelial growth factor.
